# Agreement and prognostic accuracy of three ED vulnerability screeners: findings from a prospective multi-site cohort study

**DOI:** 10.1007/s43678-023-00458-6

**Published:** 2023-03-01

**Authors:** Fabrice I. Mowbray, George Heckman, John P. Hirdes, Andrew P. Costa, Olivier Beauchet, Patrick Archambault, Debra Eagles, Han Ting Wang, Jeffrey J. Perry, Samir K. Sinha, Micaela Jantzi, Paul Hebert

**Affiliations:** 1 Department of Health Research Methods, Evidence, and Impact, McMaster University, Hamilton, ON Canada; 2 School of Public Health Science, University of Waterloo, Waterloo, ON Canada; 3 Schlegel Research Institute for Aging, Waterloo, ON Canada; 4 Department of Medicine and Research Center of the Geriatric University Institute of Montreal, University of Montreal, Montreal, QC Canada; 5 Division of Geriatric Medicine, Department of Medicine, Sir Mortimer B. Davis Jewish General Hospital and Lady Davis Institute for Medical Research, McGill University, Montreal, QC Canada; 6 Lee Kong Chian School of Medicine, Nanyang Technological University, Singapore, Singapore; 7 Department of Family Medicine and Emergency Medicine, Université Laval, Québec, QC Canada; 8 Centre intégré de santé et de services sociaux de Chaudière-Appalaches, Sainte-Marie, QC Canada; 9 Division of Critical Care Medicine, Department of Anesthesiology and Critical Care Medicine, Université Laval, Québec City, QC Canada; 10 Department of Emergency Medicine, School of Epidemiology and Public Health, University of Ottawa, Ottawa, ON Canada; 11 Ottawa Hospital Research Institute, Ottawa, ON Canada; 12 Division of Critical Care Medicine, Department of Medicine, Universite de Montreal, Montreal, QC Canada; 13 Division of Geriatric Medicine, Department of Medicine, Sinai Health and University Health Network, Toronto, ON Canada; 14 Division of Geriatric Medicine, Department of Medicine, University of Toronto, Toronto, ON Canada; 15 Division of Palliative Care, Department of Medicine, Bruyere Research Institute, University of Ottawa, Ottawa, ON Canada

**Keywords:** Vulnerability, Frailty, Geriatrics, Emergency department, Screening, Vulnérabilité, Fragilité, Gériatrie, Département d'urgence, Dépistage

## Abstract

**Objectives:**

To evaluate the agreement between three emergency department (ED) vulnerability screeners, including the InterRAI ED Screener, ER^2^, and PRISMA-7. Our secondary objective was to evaluate the discriminative accuracy of screeners in predicting discharge home and extended ED lengths-of-stay (> 24 h).

**Methods:**

We conducted a nested sub-group study using data from a prospective multi-site cohort study evaluating frailty in older ED patients presenting to four Quebec hospitals. Research nurses assessed patients consecutively with the three screeners. We employed Cohen's Kappa to determine agreement, with high-risk cut-offs of three and four for the PRISMA-7, six for the ER^2^, and five for the interRAI ED Screener. We used logistic regression to evaluate the discriminative accuracy of instruments, testing them in their dichotomous, full, and adjusted forms (adjusting for age, sex, and hospital academic status).

**Results:**

We evaluated 1855 older ED patients across the four hospital sites. The mean age of our sample was 84 years. Agreement between the interRAI ED Screener and the ER^2^ was *fair* (*K* = 0.37; 95% CI 0.33–0.40); agreement between the PRISMA-7 and ER^2^ was also *fair* (*K* = 0.39; 95% CI = 0.36–0.43). Agreement between interRAI ED Screener and PRISMA-7 was *poor* (*K* = 0.19; 95% CI 0.16–0.22). Using a cut-off of four for PRISMA-7 improved agreement with the ER^2^ (*K* = 0.55; 95% CI 0.51–0.59) and the ED Screener (*K* = 0.32; 95% CI 0.2–0.36). When predicting discharge home, the concordance statistics among models were similar in their dichotomous (*c* = 0.57–0.61), full (*c* = 0.61–0.64), and adjusted forms (*c* = 0.63–0.65), and poor for all models when predicting extended length-of-stay.

**Conclusion:**

ED vulnerability scores from the three instruments had a fair agreement and were associated with important patient outcomes. The interRAI ED Screener best identifies older ED patients at greatest risk, while the PRISMA-7 and ER^2^ are more sensitive instruments.

**Supplementary Information:**

The online version contains supplementary material available at 10.1007/s43678-023-00458-6.

## Résumé

**Objectifs:**

Évaluer la concordance entre trois outils de dépistage de la vulnérabilité des urgences, notamment l'InterRAI ED Screener, ER^2^ et PRISMA-7. Notre objectif secondaire était d'évaluer la précision discriminative des agents de dépistage dans la prédiction de la sortie à domicile et des durées de séjour prolongées à l'urgence (> 24 heures).

**Méthodes:**

Nous avons mené une étude de sous-groupe emboîtée à partir des données d'une étude de cohorte prospective multi-sites évaluant la fragilité chez les patients plus âgés se présentant aux urgences de quatre hôpitaux québécois. Les infirmières de recherche ont évalué les patients consécutivement avec les trois dépisteurs. Nous avons utilisé le Kappa de Cohen pour déterminer la concordance, avec des seuils de risque élevé de trois et quatre pour le PRISMA-7, de six pour l'ER^2^ et de cinq pour l’ interRAI ED Screener. Nous avons utilisé la régression logistique pour évaluer la précision discriminante des instruments, en les testant dans leur forme dichotomique, complète et ajustée (en ajustant pour l'âge, le sexe et le statut académique).

**Résultats:**

Nous avons évalué 1 855 patients âgés aux urgences dans les quatre sites hospitaliers. L'âge moyen de notre échantillon était de 84 ans. La concordance entre l'interRAI ED Screener et l'ER^2^ était *équitable* (K =0,37 ; IC à 95 % =0,33-0,40) ; la concordance entre le PRISMA-7 et l'ER^2^ était également *équitable* (K = 0,39 ; IC à 95 % =0,36-0,43). La concordance entre interRAI ED Screener et PRISMA-7 était *faible* (K = 0,19 ; IC à 95 % = 0,16-0,22). L'utilisation d'un seuil de quatre pour PRISMA-7 a amélioré la concordance avec l'ER^2^ (K =0,55 ; IC à 95% =0,51-0,59) et l'ED Screener (K =0,32 ; IC à 95 % =0,2-0,36). En ce qui concerne la prédiction du retour à domicile, les statistiques de concordance entre les modèles étaient similaires dans leurs formes dichotomiques (c = 0,57-0,61), complètes (c =0,61-0,64) et ajustées (c =0,63-0,65), et faibles pour tous les modèles en ce qui concerne la prédiction de la durée de séjour prolongée.

**Conclusion:**

Les scores de vulnérabilité aux urgences des trois instruments concordaient assez bien et étaient associés à des résultats importants pour les patients.

## Clinician’s capsule

**Table:** 

***What is known about the topic?***
Little is known about the agreement or prognostic value among the InterRAI ED Screener, ER^2^, and PRISMA-7 in older ED patients.
***What did this study ask?***
Do these instruments agree in their classification of high-risk older ED patients and can they predict ED outcomes?
***What did this study find?***
All instruments could predict the need for hospitalization but performed poorly when predicting LOS. Instruments had fair agreement, and the ED Screener was most specific at predicting ED outcomes.
***Why does this study matter to clinicians?***
We provide data to support decision-making about which ED vulnerability screener to consider to best meet the needs of older patients and ED providers.

## Introduction

Emergency departments (ED) are caring for an ever-increasing number of older adults presenting with complex medical and social needs [1]. Acuity-driven emergency management pathways habitually overlook the intricate and patient-specific needs of older ED patients [2, 3]. To improve the patient experience and health outcomes of older ED patients, geriatric emergency guidelines and experts have emphasized the importance of proactively targeting patients at risk for adverse health events, and thus in greatest need of a detailed geriatric assessment [4–6]. Comprehensive geriatric assessment is the gold standard approach when evaluating older adults. However, its administration may be difficult in the dynamic and fast-paced environment of the ED where staffing, resources, and medical acuity, are continuously changing [6–8].

Utilizing vulnerability screeners in the ED has been proposed as a pragmatic method to rapidly identify high-risk older patients for early intervention in the emergency management pathway [6]. Geriatric syndromes are commonly overlooked by ED clinicians and acuity-driven emergency models of care [9, 10], though they are more likely to be identified when using a vulnerability screener [11]. Thus, vulnerability screeners are used to identify and triage patients with high-risk geriatric syndromes (e.g., frailty, functional or cognitive impairment) that increase the risk of adverse health events [6, 12, 13].

Several ED vulnerability screeners have been derived and validated, with the interRAI ED Screener [14, 15], Emergency Room Evaluation and Recommendations (ER^2^) [16–18], and the Program of Research on Integration of Services for the Maintenance of Autonomy (PRISMA-7) [19–21] most commonly used in Canadian ED settings. These screeners have also been implemented and appraised in older ED populations internationally [15, 16, 20, 22–27]. The interRAI ED Screener, ER^2^, and PRISMA-7 prioritize and evaluate several shared geriatric syndromes, though little is known about the agreement between these instruments in classifying patients as 'high-risk.' Foreknowledge of geriatric complexity can support decision-making for clinicians and policymakers about which vulnerability screener to consider and which cluster of assessment items can best support targeted referrals to geriatric services from the ED, given the limited resources available.

In this study, we proposed to determine the level of agreement between the InterRAI ED Screener, ER^2^, and PRISMA-7. We also compared the discriminative accuracy of these instruments in predicting two patient-important outcomes, discharge to home and extended lengths of stay (LOS) in the ED.

## Methods

### Study design and setting

We conducted a nested sub-group study using data from a prospective multi-province cohort study evaluating frailty in older ED patients. Originally, study sites were spread between Ontario, Quebec, and Newfoundland, and all sites administered an ED vulnerability screener. For this study, we only evaluated data from Quebec hospitals, as they were the only sites to administer all three ED vulnerability screening instruments to eligible patients. Patients aged 65 years and older were consecutively screened for study eligibility by research staff during ED registration, based on a pre-defined study enrolment period of January 1, 2017–July 31, 2018. We obtained ethics approval from the research ethics boards of all participating hospital sites and the University of Waterloo. We used the Strengthening the Reporting of Observational Studies in Epidemiology (STROBE) statement to guide the reporting of this study [28].

### Population

We recruited patients for the study during daytime hours (0700–1900) when older adults are more likely to present for emergency care [29]. Older adults were identified using ED rosters upon the start of the day shift (0700 h). Research staff completed the three instruments during the triage process or within a few hours of admission to the ED. We excluded patients determined to be medically unstable or in need of emergent care or resuscitation, leveraging the clinical decision-making of the triage nurse. We also excluded patients if they, or their caregivers, could not answer questions in either French or English. We obtained consent to participate from the patient or their substitute decision-maker.

### Intervention: ED vulnerability screening

Research nurses assessed patients with the three vulnerability screeners after receiving standardized training on the instruments and supplementary software, where necessary. They also collected data on demographics and patient outcomes. Data from the vulnerability screeners were not made available to the clinical staff. Thus, risk scores from the instruments did not influence emergency management pathways or clinical decision-making. Nurses were neither trained to evaluate agreement statistics nor responsible for this task.

### InterRAI ED Screener

The interRAI ED Screener is part of a multi-stage assessment system whereby ED screener findings inform the need for additional evaluation with a comprehensive geriatric assessment in-hospital or following discharge [6]. The interRAI ED Screener is designed to identify and triage older adults most likely to benefit from a secondary detailed geriatric assessment (e.g., interRAI ED Contact Assessment) and can be completed within minutes [14]. The interRAI ED Screener includes questions regarding activities of daily living (ADLs), cognitive skills for daily decision-making, self-rated mood and health, caregiver distress, unstable medical condition, and dyspnea [14]. The ED Screener is sequenced and adapted based on prior item responses, with most high-risk persons identified within a few screening questions [14], and scores range from one to six. The presence of any functional impairment automatically scores patients as moderate risk (≥ 4), with priority further driven by indicators of caregiver distress, low mood and poor self-hygiene [14]. The interRAI ED Screener is free, readily available (www.interRAI.org), and has proven to be predictive of patient-important outcomes, including mortality, lengthy hospital stays, and the need for more formal support following discharge [15, 22, 30].

### PRISMA-7

The PRISMA-7 is designed to identify older adults with disabilities and can be completed within a few minutes [19, 20, 27]. The instrument has been reported to accurately classify frail ED patients compared to the results of a comprehensive geriatric assessment and is suitable for completion by emergency nurses and physicians [27, 31]. The PRISMA-7 consists of seven questions examining the patient's age, sex, limitations with activities of daily living or travel outside of the house, the need for regular support, the presence of a dependable support system, and the use of locomotive assistive devices (e.g., cane) [19]. The number of positive items flagged is summed to create a final score ranging from zero to seven. Scores of zero to two are classified as low-risk, and scores of three and greater as high-risk [19]. The PRISMA-7 has been widely accepted and utilized among many Quebec hospitals to evaluate high-risk older ED patients [32].

### ER^2^

The ER^2^ is a brief questionnaire designed to identify older ED patients at high risk of short-term adverse events (e.g., ED length-of-stay, hospital admission). In prior evaluations, the instrument took less than three minutes to complete [16, 18]. A high-risk classification and a positive flag for temporal disorientation are closely linked with the presence of a major neurocognitive disorder [17]. The ER^2^ consists of six closed-ended questions about the patient's age, sex, the need for support in the home, polypharmacy, the use of a walking aid, and the ability to identify the current year and month correctly [16]. The latter two items carry five times the weight when positive, resulting in a scale ranging from zero to fourteen. The authors report that a score of zero to three is classified as low risk, four and five as moderate risk, and six or greater as high risk [16]. We elected to include the ER^2^, given its evaluation of well-established geriatric syndromes and the recent development and implementation of this instrument within Quebec hospital systems [17].

### Outcome measures

The primary outcome of our study was a high-risk score from any of the three ED vulnerability screeners. Specifically, we were interested in the overall agreement between the three screeners in classifying high and low-risk patients. We calculated agreement in a post-hoc fashion, given the secondary nature of this study. We re-classified outputs from all instruments into 'high' and 'not high-risk' categories to account for the lack of a 'moderate risk' stratification group for the PRISMA-7. We defined high-risk using the cut-offs reported originally during instrument derivation [14, 16, 19]. Secondary outcomes for this study included discharge-to-home and an extended ED length-of-stay (> 24 h). We elected to use this cut-off as it conceptually represents a full day of care in the ED, is common among older ED patients, and better signals high-risk older ED patients than a 12-h cut-off [33]. ED registration time was used as the point of reference when determining ED length-of-stay, and discharge time as the end-point. Both discharge location and ED length-of-stay are a priority concern for patients [34, 35].

### Data analysis

We reported descriptive statistics of all baseline characteristics using general frequency and central tendency measures. We evaluated instrument agreement using Cohen's Kappa. We utilized logistic regression to determine the discriminative ability of the three screener scores in predicting patient outcomes in their dichotomous, full, and statistically adjusted forms. Truncated models included vulnerability screeners using the cut-offs proposed by the original authors. Specifically, we used a cut-off of five or greater for the interRAI ED Screener, six or greater for the ER^2^, and three or greater for the PRISMA-7. We also conducted a sensitivity analysis testing agreement with the PRIMSA-7 using a cut-off of four or greater, mindful that many hospitals in Quebec use this cut-off to support decision-making and patient trajectories. Full-form models used the entire range of screener scores. Building on full-form models, adjusted models controlled for patient age, sex and academic status of the treating institution. Concordance statistics and the corresponding confidence intervals are reported for all models. We evaluated the statistical significance of differing concordance statistics using methods proposed by DeLong [36]. Cases with missing data were deleted within each analysis. We managed and analyzed data using the *stats, psych, and pROC* packages in **R** (version 4.0.0).

## Results

**Fig. 1:**
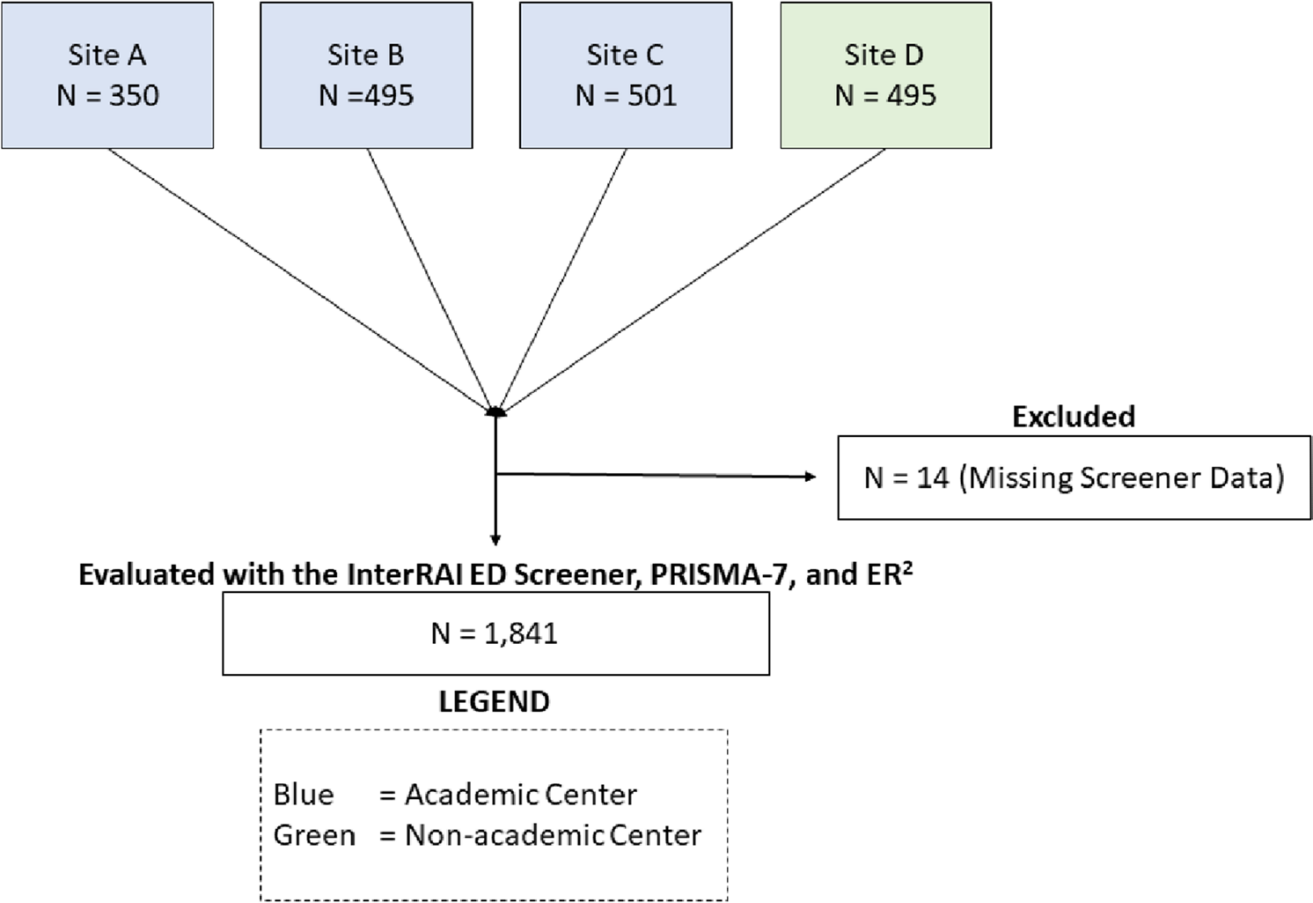
Flow diagram of vulnerability screener use across study sites

A total of 1855 older ED patients were evaluated across the four hospital sites using all three vulnerability screeners. The mean age of the sample was 84 years, and most participants were female (58%). The mean LOS in the ED was 20.1 h, and many were classified as high-risk using the interRAI ED Screener (38.3%), PRISMA-7 (83.2%), and ER^2^ (59.1%). Over one-third of patients were discharged home from the ED (36%) or experienced an extended LOS in the ED (37%). Table 1 displays the patient profiles and screener scores assigned. The distribution of vulnerability screener scores can be seen in their full un-truncated form in the supplementary file. Missing data was only present for outcome data, though it was scant (< 1%). A flow diagram of all patients evaluated can be found in Fig. 1.

**Table 1:** Baseline characteristics for 1855 older ED patients evaluated in Quebec, Canada with interRAI ED Screener, ER^2^, and PRISMA-7

Prognostic factors	*N* (%)
Age^a^	84 (5.8)
Gender (Female)	788 (57.5)
Daytime presentation^b^	1795 (97.2)
Study site	
A	355 (19)
B	499 (26.9)
C	502 (27.2)
D (No Academic Affiliation)	499 (26.9)
interRAI ED Screener Score	
0–2	430 (23.2)
3–4	715 (38.5)
5–6 (High-Risk)	710 (38.3)
PRISMA-7 Score	
0–2	329 (17.7)
3 + (High-Risk)	1526 (82.3)
ER^2^ Score	
0–3	694 (37.4)
4–5	64 (3.5)
6 + (High-Risk)	1097 (59.1)
Length of ED Stay (Hours)^a^	20.1 (11.8)

^a^Reported as mean (standard deviation)

^b^Patient presented to the emergency department between 0700 and 1900 h

Full granularity of the distribution pattern for vulnerability screeners can be found in Online Appendix A

### Agreement

**Fig. 2:**
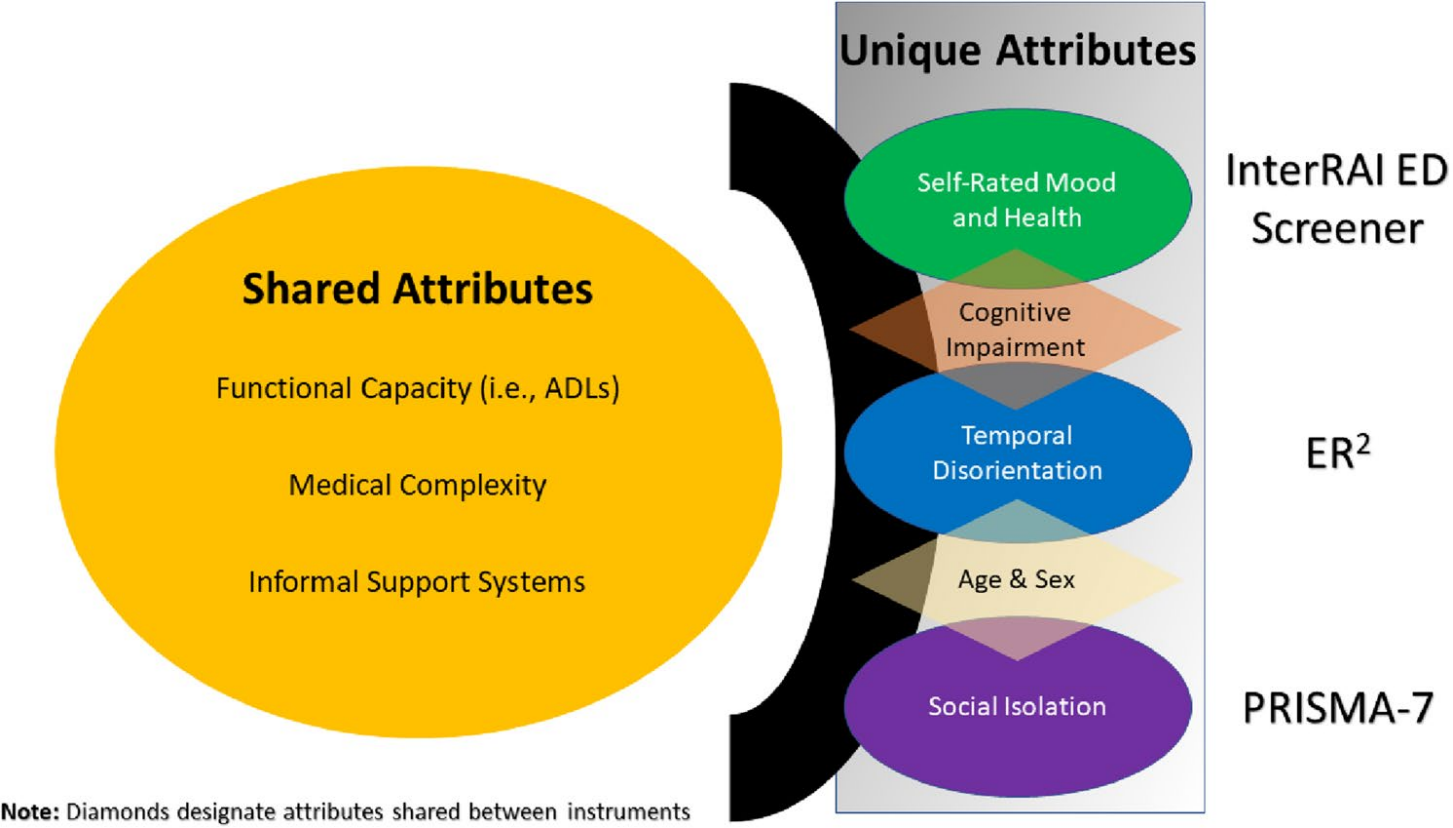
Shared and unique attributes among the interRAI ED Screener, ER^2^, and PRISMA-7

Tables 2, 3 and 4 display the cross-tabulations of vulnerability screener scores and their agreement when classifying those at high-risk (i.e., Cohen's Kappa values). The overall agreement between the interRAI ED Screener and the ER^2^ was *fair* (*K* = 0.37; 95% CI 0.33–0.40). A comparable level of agreement between the PRISMA-7 and the ER^2^ was also noted (*K* = 0.39; 95% CI 0.36–0.43), though improved when using a cut-off of four or greater for the PRISMA-7 (*K* = 0.55; 95% CI 0.51–0.59). The agreement between interRAI ED Screener and the PRISMA-7 was *poor* using a cut-off of three or greater for the PRISMA-7 (*K* = 0.19; 95% CI 0.16–0.22). However, when using a cut-off of four or greater, the agreement between these two instruments improved (*K* = 0.32; 95% CI 0.29–0.36). Figure 2 displays the unique and shared attributes among vulnerability screeners used to classify high-risk patients.

**Table 2:** Crosstabulation of agreement between 1855 older ED patients assessed with the interRAI ED Screener and ER^2^

interRAI ED Screener	ER^2^		Total
	Not high risk (Score 0–5)	High-risk (Score 6 +)	
Not high risk (Score 1–4)	645 (34.8%)	500 (27%)	1145
High risk (Score 5–6)	113 (6.9%)	597 (32.1%)	710
Total	759	1097	1855

Proportions are of the total sample. Kappa = 0.37 (95% Confidence interval = 0.33–0.4)

**Table 3:** Crosstabulation of agreement between 1855 older ED patients assessed with the interRAI ED Screener and PRISMA-7

interRAI ED Sscreener	PRISMA-7		Total
	Not high risk (Score 0–2)	High-risk (Score 3 +)	
Not High Risk (Score 1–4)	305 (16.4%)	840 (45.2%)	1145
High Risk (Score 5–6)	24 (1.3%)	686 (37%)	710
Total	329	1526	1855

Proportions are of the total sample. Kappa = 0.19 (95% Confidence interval = 0.16–0.22)

**Table 4:** Crosstabulation of agreement between 1855 Older ED patients assessed with the PRISMA-7 and ER^2^

ER^2^	PRISMA-7		Total
	Not high risk (Score 0–2)	High-risk (Score 3 +)	
Not high risk (Score 0–5)	296 (16%)	462 (24.9%)	758
High Risk (Score 6 +)	33 (1.8%)	1064 (57.4%)	1097
Total	329	1526	1855

Proportions are of the total sample. Kappa = 0.39 (95% Confidence interval = 0.36–0.43)

### Discriminative ability of vulnerability screeners

Table 5 displays the discriminative accuracy of all models in their dichotomous, full, and adjusted forms.

**Table 5:** Discriminative accuracy of three ED vulnerability screeners in predicting discharge home and extended ED length of stay in 1841 older ED patients

	Discharge home			Extended ED length of stay		
Instrument	Truncated screener	Full screener	Adjusted screener	Truncated screener	Full screener	Adjusted screener
interRAI ED Screener	0.61 (0.58–0.63)	0.62 (0.59–0.65)	0.64 (0.62–0.67)	0.53 (0.51–0.55)	0.54 (0.51–0.56)	0.55 (0.52–0.57)
ER^2^	0.60 (0.58–0.63)	0.61 (0.59–0.64)	0.63 (0.60–0.65)	0.52 (0.49–0.54)	0.54 (0.52–0.57)	0.55 (0.52–0.58)
PRISMA-7	0.57 (0.55–0.58)	0.64 (0.61–0.66)	0.65 (0.62–0.67)	0.51 (0.49–0.53)	0.52 (0.5–0.56)	0.54 (0.52–0.57)

All data is presented as concordance statistics (95% confidence intervals)

Adjusted models controlled for age, sex, and academic affiliation of the hospital

### Discharge Home

The discriminative ability of the three ED vulnerability screeners were similar when evaluated in their intended form (*c* = 0.57–0.61), full form (*c* = 0.61–0.64), and adjusted form (*c* = 0.63–0.65). These models predicted hospital discharge with *fair* accuracy. There was no significant difference in model accuracy between the three instruments (*p* > 0.05). Similarly, assessing the models in their full and adjusted form did not significantly improve the discriminative accuracy of statistical models.

In adjusted models, the academic status of the treating institution significantly increased the odds of a discharge home in models evaluating the interRAI ED Screener (OR = 1.59; 95% CI 1.26–2.0) and the PRISMA-7 (OR = 1.46; 95% CI 1.1–1.75). Similar results were found for the ER^2^ (OR = 1.42; 95% CI 1.13–1.79). Male patients were also more likely to be discharged home when using the PRISMA-7 (OR = 1.46; 95% CI 1.17–1.8) and ER^2^ (OR = 1.28; 95% CI 1.02–1.6), the only screeners that evaluate sex.

### Extended ED length-of-stay (> 24 h)

The discriminative accuracy of all three models was poor when predicting ED length-of-stay. All screeners had similar performance metrics, with concordance statistics ranging from 0.51 to 0.55. The adjusted model examining the discriminative accuracy of the interRAI ED Screener was the only multivariable model to note a significant association between academic status and a decreased risk for an extended LOS in the ED (OR = 0.78; 95% CI 0.62–0.97).

## Discussion

### Interpretation

When comparing ED vulnerability scores with one another, the interRAI ED Screener, ER^2^ and PRISMA-7 had fair agreement amongst themselves, despite sharing a number of similar assessment items. ED vulnerability scores from the three instruments were associated with patient-important and clinically relevant outcomes, including discharge home and length of stay in the ED. These tools have prognostic value, despite being designed to direct individuals for more detailed assessments or towards geriatric care pathways.

### Previous studies

Few, if any, studies examined agreement between the three ED vulnerability screeners. However, the age and sex distributions of our study were similar to other studies evaluating the use of vulnerability screeners in the ED, with most studies having mean ages over 80 years and a greater proportion of female patients. Prior studies of agreement found the interRAI ED Screener demonstrates fair-to-good agreement in classifying high-risk older ED patients when compared with the Acutely Presenting Older Patient (APOP) screener and Identification of Seniors at Risk (ISAR) screener [37]. When using a comprehensive geriatric assessment as the reference standard to classify frail patients, the PRISMA-7 performed better but similar to the ISAR and the Clinical Frailty Scale when discriminating patients with frailty [31]. Our study supports prior work demonstrating a positive association between high-risk screener classifications, hospital admission and an extended ED LOS, when using the interRAI ED Screener [15, 30], PRISMA-7 [20], or ER^2^ [16, 21]. Our study differs in our use of a longer 24-h cut-off to define an extended stay. However, this likely promotes the external validity of our findings to other Canadian provinces where ED wait times are known to be lengthy [38, 39].

### Strengths and limitations

A key strength of our study was the inclusion of multiple and diverse sites actively involved in recruiting and evaluating patients. In addition to academic and community sites with a variety of referral patterns in several health systems, we also included a wide variety of patients including older adults who presented with cognitive impairment, a cohort commonly excluded in clinical and emergency research.

We also note some limitations. We were pragmatic and cost constrained in our approach, so minimal clinical data were collected. Data on presenting complaints, diagnoses, and triage acuity would likely have enriched our analysis. Additionally, data on mortality were not collected, though prior work has demonstrated than less than 1% of older Canadian patients will die in the ED [40], and approximately 5% will die within one month of presentation [41, 42]. Finally, since we used ED discharge as the end-point for the patient journey rather than ED disposition decision, factors related to ED and hospital system care may confound our understanding of the association between vulnerability screeners and LOS.

### Clinical implications

All three vulnerability screeners had similarities and key differences in their operating characteristics. When we examine disagreements, we note that the interRAI ED Screener classified 27% of individuals as low risk, while the ER^2^ classified them as high risk. When compared to the PRISMA-7 score, interRAI classified 45% of individuals who were considered high-risk based on PRISMA-7 scores. Thus, if ED care providers hope to identify the highest-risk individuals, then the interRAI ED Screener would be the best choice. If, on the other hand, ED care providers hope to use a more sensitive instrument, then using PRISMA-7 and to a lesser extent the ER^2^ would seem most appropriate. These differences may be a result of slight differences in questions, or the fact that the interRAI ED Screener is a response-adaptive algorithm.

Our study found all vulnerability screeners were able to classify those in need of hospital admission with fair accuracy even after adjusting for age, sex, and academic status of the treating institution. It is worth noting that only the ER^2^ was the only instrument specifically purposed to predict health service outcomes. Given the complexity of causal pathways for disposition and length of stay, it is noteworthy if any positive association is detected. These associations justify the need to identify high-risk individuals, propose detailed assessments, and consider care paths to ensure a timely and efficient response to complex geriatric syndromes and unmet social needs.

### Research implications

From our work, we believe further work is required to understand how screening strategies may best be included in care pathways that make use of more detailed geriatric assessments or consultations from geriatric specialists. In addition, further work may examine whether screening strategies may be used to guide interventions designed to decrease admission rates or mitigate the consequences of ED visits.

## Conclusion

Our study demonstrated an association between all included ED vulnerability screeners and patient-important outcomes. Thus, any of these three ED vulnerability screeners can support the triage and care of high-risk older adults in the ED. However, the interRAI ED Screener classified more individuals as low risk compared to the ER^2^ and PRISMA-7. Agreement among instruments improved using a PRIMSA-7 cut-off of four or greater. Our research supports the implementation of ED screening tools.

## Supplementary Information

Below is the link to the electronic supplementary material.

Supplementary file1 (DOCX 28 KB)

## Data Availability

Data generated during this study are not publicly available. The datasets generated and analysed during the current study are not publicly available because they contain information that could compromise research participant privacy/consent but are available from Dr. John Hirdes on reasonable request.
