# Supplementary material for: Agreement and prognostic accuracy of three ED vulnerability screeners: findings from a prospective multi-site cohort study
Source: CJEM. 2023 Mar 1;25(3):209–17. doi: 10.1007/s43678-023-00458-6 (PMC10014815; doi:10.1007/s43678-023-00458-6)
Supplement: Supplementary file 1 — Supplementary file1 (DOCX 28 KB) [file 43678_2023_458_MOESM1_ESM.docx]

**Appendix A**

Baseline Characteristics for 1,855 Patients Evaluated with the
interRAI ED Screener, PRISMA-7 and the ER^2^

| **Vulnerability Screener** | **N (%)** |
| --- | --- |
| **Assessment Urgency Algorithm Score**  **1** (Least Urgent)  **2**  **3**  **4**  **5**  **6** (Most Urgent) | 350 (18.9)  80 (4.3)  475 (25.6)  240 (12.9)  70 (3.8)  640 (34.5) |
| **PRISMA-7 Score**  **0** (Least Urgent)  **1**  **2**  **3**  **4**  **5**  **6**  **7** (Most Urgent) | 10 (0.5)  76 (4.1)  243 (13.2)  287 (15.6)  384 (20.9)  411 (22.3)  340 (18.4)  90 (4.9) |
| **ER2 Score**  **0** (Least Urgent)  **1**  **2**  **3**  **4**  **5**  **6**  **7**  **8**  **9**  **10**  **11**  **12**  **13**  **14** (Most Urgent) | 41 (2.2)  173 (9.2)  316 (17.1)  164 (8.9)  44 (2.4)  20 (1.1)  83 (4.5)  320 (17.1)  410 (22.2)  120 (6.5)  1 (0.1)  15 (0.8)  45 (2.4)  79 (4.2)  24 (13) |
